# Case Report: From epilepsy and uterus didelphys to Turner syndrome-associated dysgerminoma

**DOI:** 10.3389/fgene.2023.1286515

**Published:** 2024-01-11

**Authors:** Jinghua Li, Haipeng Zhu, Xuelian Ma, Jia Li, Jing Xue, Limin Feng

**Affiliations:** ^1^ Department of Obstetrics and Gynecology, Beijing Tiantan Hospital, Capital Medical University, Beijing, China; ^2^ Centre for Personalized Cancer Therapy, Ciming Boao International Hospital, Lecheng International Medical Tourism Pilot Zone, Qionghai, Hainan, China; ^3^ P&A Consulting, Adelaide, SA, Australia; ^4^ Department of Pathology, Beijing Tiantan Hospital, Capital Medical University, Beijing, China; ^5^ Department of Radiology, Beijing Tiantan Hospital, Capital Medical University, Beijing, China

**Keywords:** epilepsy, uterus didelphys, Turner syndrome, dysgerminoma, KIT mutation

## Abstract

Dysgerminoma is a rare occurrence in Turner syndrome patients without Y chromosome mosaicism or hormone therapy during puberty. We present a unique case of a 33-year-old nulliparous Chinese woman with intermittent epilepsy and Mullerian anomalies carrying a double uterus, cervix, and vagina. The patient is also characterized as having Turner syndrome accompanied by 46,X, del(Xp22.33-11.23) and del(2)(q11.1-11.2). MRI exhibited a 17.0 cm × 20.0 cm × 10.5 cm solid ovarian lesion. Radical surgery and pathology revealed dysgerminoma at stage IIIc with lymphatic metastases and a KIT gene mutation identified in exon 13. Furthermore, the tumor microenvironment (TME) displayed robust expression of CD4^+^ T lymphocytes and PD-1, whereas the distribution of CD8^+^ T lymphocytes and PDL-1 was sporadic. Despite the administration of enoxaparin to prevent thromboembolism, the patient experienced multiple cerebral infarctions during chemotherapy. Subsequently, the patient chose to decline further treatment and was discharged. This exceptional case imparts several noteworthy lessons. First, the coexistence of Mullerian anomalies, although rare, is not incompatible with Turner syndrome. Second, screening for KIT mutations is imperative to reduce the risk of dysgerminoma in Turner syndrome, especially for patients with Y mosaicism who are recommended for hormone replacement therapy. Lastly, comprehensive anticoagulation therapy is crucial for Turner syndrome patients undergoing cisplatin-based chemotherapy.

## Introduction

Turner syndrome encompasses a range of clinical manifestations tightly linked to various X chromosome abnormalities, particularly 45,X, 45,X/46,XY, and 46,X,del(Xp) (Murdock et al., 2017). Recent advances in genetic sequencing techniques have enabled the accurate identification of numerous Turner syndrome karyotypes including 46,X,del(Xp) (Prakash et al., 2014; Murdock et al., 2017). While Turner syndrome occupies many a phenotype, the coexistence of a double vagina, cervix, and uterus is unprecedented (Vaddadi et al., 2013). Furthermore, dysgerminoma occurs in only 1% of Turner syndrome patients without Y mosaicism (Roth et al., 2019), whereas only 2% of Turner syndrome genotypes showed 46,X,del(Xp) (Sybert and McCauley, 2004). In addition, being a tyrosine kinase receptor, the mutant KIT gene plays a pivotal role in carcinogenesis through multiple molecular pathways (Abdellateif et al., 2023). The mutations of the KIT gene are mainly identified in gastrointestinal stromal tumors in exons 8, 9, 11, 13, and 17 (McAuliffe et al., 2008), whereas human germ cell tumors and testicular seminomas contain the mutations in exons 11 and 17 (Hersmus et al., 2012). Currently, there is no evidence showing a mutation of the KIT gene in exon 13 existing in human germ cell tumors (Hersmus et al., 2012).

## Case report

A 33-year-old nulliparous woman presented with severe lower abdominal pain was admitted to the Emergency Department in March 2023. The patient had a history of mild hypertension and intermittent epilepsy since the age of 23 years and was treated with oxcarbazepine for 10 years. The patient experienced menarche at the age of 16 years, followed by irregular periods starting at the age of 19 years, and subsequently developed amenorrhea from the age of 23 years onward. The patient showed normal intelligence and social communication, and no history of hormone therapy was documented. Physical examination showed normal breast development and absence of pubic hair growth. The patient’s height was 150 cm and weight was 45 kg. Moreover, an irregularly palpated mass was found in the lower left abdomen, extending to the umbilicus, accompanied by a double vagina and cervix. Blood tests revealed elevated platelet (380*10E9/L), LDH (300.5 U/L), and β-hCG (24.45 mIU/mL) levels, while AFP, CEA, CA-125, and CA-19-9 levels were within the normal range. Additionally, the sexual hormone panel and coagulative file were unremarkable. Except for hyperlipidemia, functional tests of the thyroid, liver, and kidneys were normal. After radical surgery, LDH and β-hCG levels returned to the normal levels at 194.4 U/L and 1.32 mIU/mL, respectively.

**FIGURE 1:**
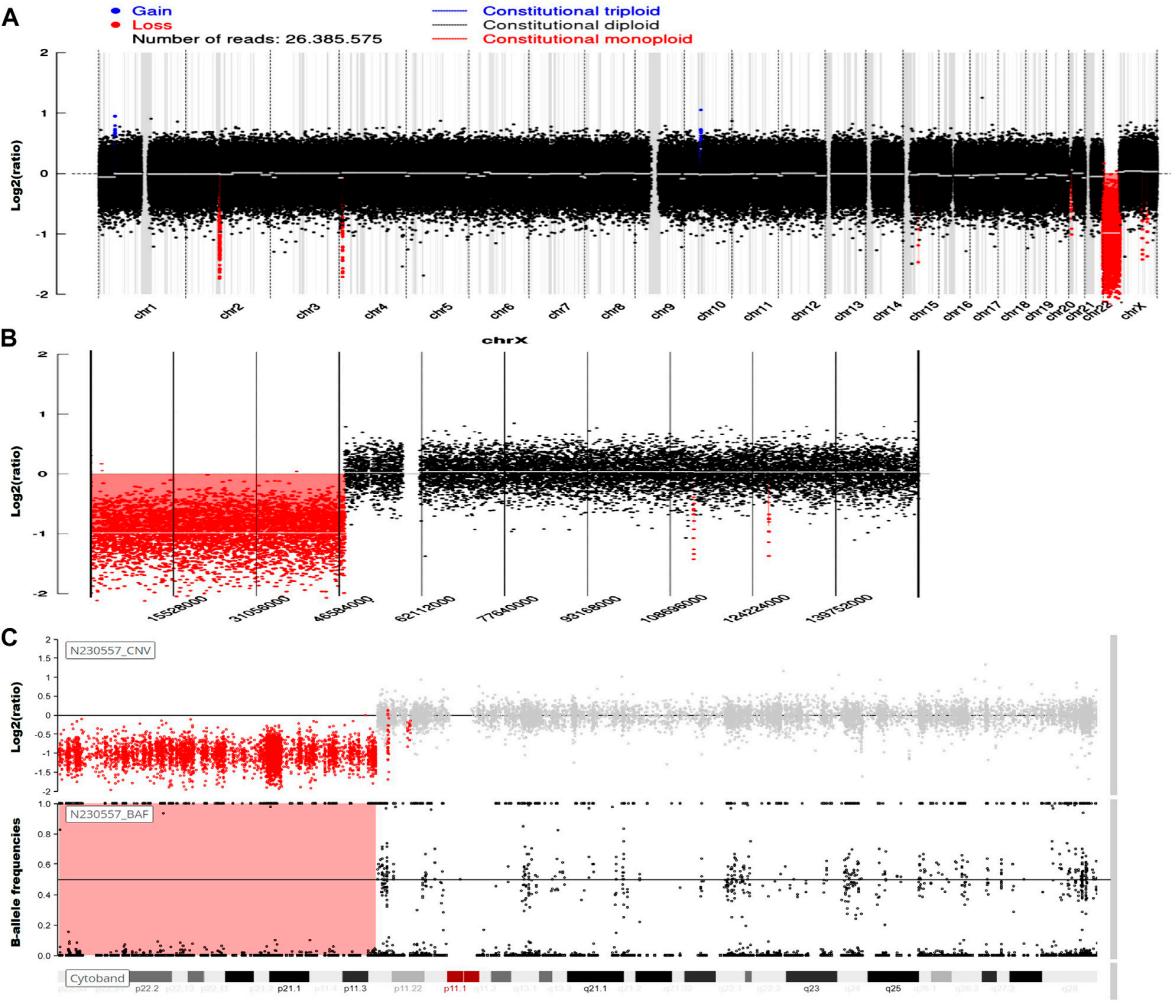
Genetic analysis.

**FIGURE 2:**
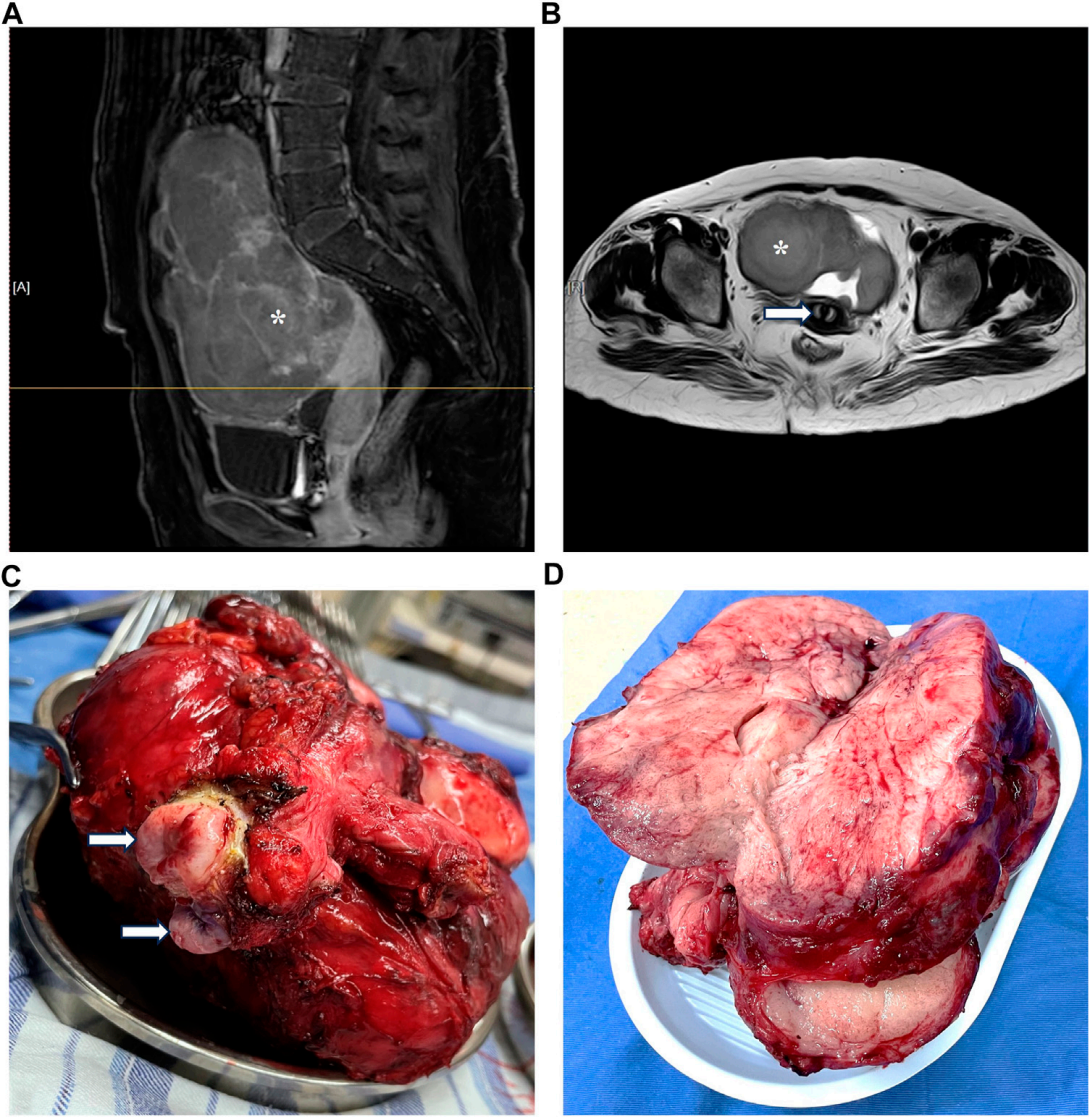
Magnetic resonance imaging and radical surgery.

Genetic testing in March 2023 revealed the presence of 46,X,del(Xp22.33-11.23), involving a 47.6-Mb deletion, along with del(2)(q11.1-11.2), characterized by a 1.5-Mb deletion (Figure 1). An MRI investigation also exhibited a 17.0 cm × 20.0 cm × 10.5 cm solid ovarian lesion and uterus didelphys (Figure 2), whereas cranial and pulmonary CT examinations yielded unremarkable findings. Echocardiography confirmed slight tricuspid and mitral regurgitation.

**FIGURE 3:**
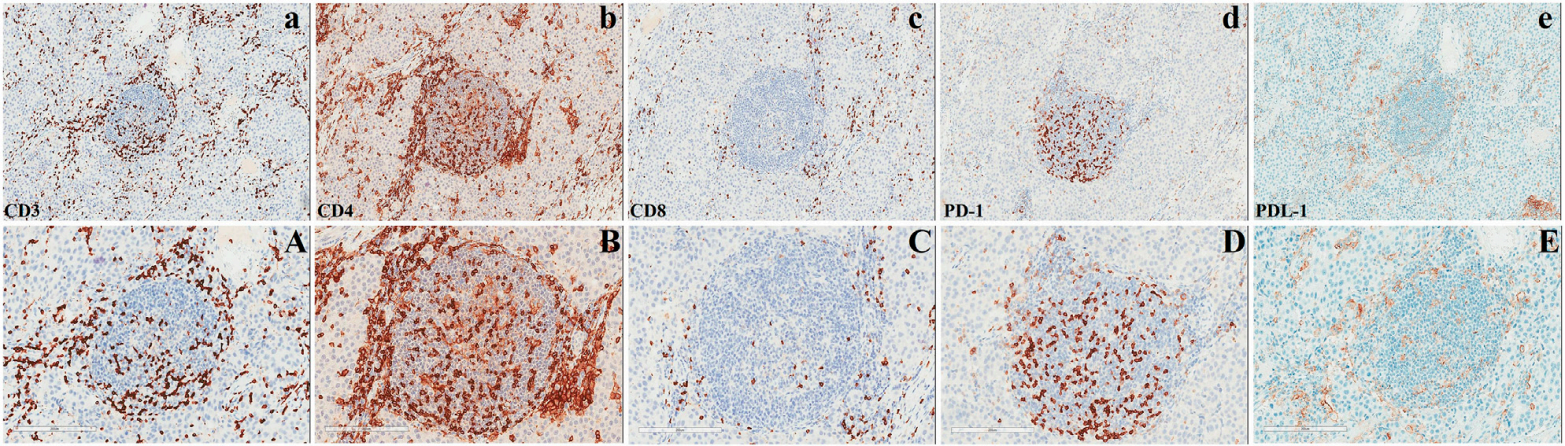
Analysis of the microenvironment in dysgerminoma.

In March 2023, the patient underwent radical surgery, in which pathology verified dysgerminoma at stage IIIc with lymphatic metastases (Figure 2). A cancer-associated mutation screening program utilizing dysgerminoma tissue identified the N655K mutation within exon 13 of the KIT gene. Subsequent immunohistochemistry analysis of sequentially sectioned tumor slides demonstrated the robust expression of CD4^+^ T cells and programmed cell death protein 1 (PD-1) within the tumor microenvironment (TME), whereas staining of CD8^+^ T cells and programmed death-ligand 1 (PDL-1) showed sporadic distribution (Figure 3).

**FIGURE 4:**
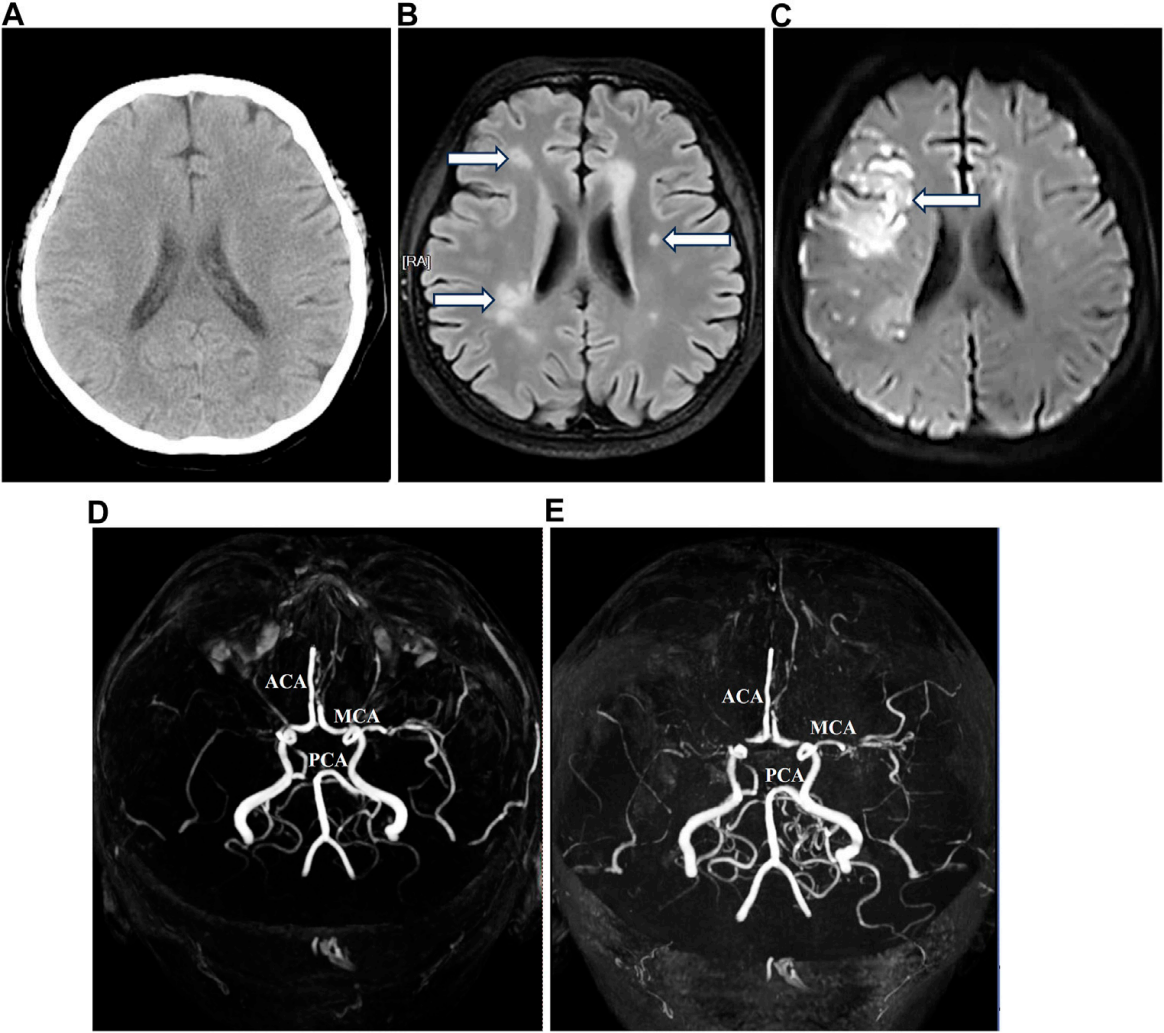
Magnetic resonance imaging and magnetic resonance angiography before and after chemotherapy.

After radical surgery, the patient experienced a single episode of spontaneous cerebral infarction at home in April 2023, which was treated at another tertiary hospital with complete recovery. Before the onset of chemotherapy, an evaluation at our hospital in May 2023 via intracranial MRI and MRA exhibited previously infarcted foci, highly suspected metastatic loci, and the circle of Willis malformation (Figure 4). Despite the daily administration of enoxaparin at a dose of 4,000 IU (40 mg) to prevent thromboembolism, multiple intracranial infarctions reoccurred (Figure 4) 5 days after commencing the standard chemotherapy regime using bleomycin, etoposide, and cisplatin. Following the comprehensive assessment and subsequent discussion of the situation, the patient chose to decline further treatment and was discharged.

## Discussion

Previously, a large segmental deletion of the Xp22.3 region in Turner syndrome patients was tightly associated with phenotypes of strabismus, ichthyosis, chondrodysplasia punctata, amenorrhea, hyperlipidemia, obesity, short stature, intellectual disability, and Kallmann syndrome due to the loss of ANOS1, GPR143, HDHD1, NLGN4X, PNPLA4, SOX10, STS, and VCX-A genes principally (Nagai et al., 2017; Ma et al., 2020). In this study, including short stature and amenorrhea, our patient also had intermittent epilepsy. Epilepsy in Turner syndrome is less common, with 2%–3% morbidity, which predominantly correlates with structural malformation in the brain and mosaicism karyotypes (Hanew et al., 2018). To the best of our knowledge, only one report of absence of epilepsy was found in a case of 46,X, del(Xp22.33) with a duplication of Xp22.32-22.12 at the age of 10 years (Puusepp et al., 2008). Intriguingly, our patient also carries a 2q11.1-11.2 deletion, which shows an increased propensity for epilepsy (Grosso et al., 2008). These findings indicate that epilepsy in this case is highly likely to manifest in early childhood, as observed in our patient at the age of 23 years after ovarian degeneration. This observation strongly suggests that estrogen plays a pivotal role in suppressing seizure generation. Furthermore, while uterus disappearance due to Mullerian agenesis has been described in the 45,X genotype, actually, most female populations with uterine and cervical malformations caused by Mullerian anomalies possess a normal pattern of sexual chromosomes (Vaddadi et al., 2013). Whether the partial or complete loss of the X chromosome acts as a selective pressure that potentially contributes to Mullerian anomalies during embryonic development, our case, the first in the world, supports an encouraging response to the 46,X,del(Xp22.33-11.23) genotype.

Previously, immunohistochemical studies of dysgerminoma showed abundant infiltrating T cells with reduced or absent expression of the major histocompatibility complex (MHC)-I and -II antigens, respectively (Saito et al., 1989; Stewart et al., 1992). Further studies demonstrated that despite the high expression of CD8^+^ T cells and PD-1/PDL-1 within the TME, immune checkpoint inhibitors (ICIs) have not yielded significant results in the treatment of refractory dysgerminoma (Adra et al., 2018; Liu et al., 2018; Boldrini et al., 2019). These investigations imply that the loss of specific cytotoxicity due to MHC-I-mediated antigen-processing deficiency in dysgerminoma directly hampers immunotherapy efficacy. Interestingly, the pathology in this case exhibited a tremendously higher expression of CD4^+^ T cells and PD-1 within the TME, whereas the staining of CD8^+^ T cells and PDL-1 showed sporadic distribution (Figure 3). Our findings suggest that CD4^+^ T cells also respond notably to the growing dysgerminoma; however, the absence of MHC-II antigens progressively causes insensitivity of CD4^+^ T cells to the tumor cells. Considering the role of CD4^+^ T cells in maintaining tumor-specific cytotoxicity of CD8^+^ T cells (Ostroumov et al., 2018) and the anticancer activity of CD4^+^ T cells against self-derived epitopes (Tay et al., 2021), enhancing MHC-II expression in dysgerminoma or infusing specific antigen-activated CD8^+^ T cells sensitized through lysates of dysgerminoma tissue *in vitro* may significantly amplify therapeutic efficacy to manage metastatic or refractory dysgerminoma.

Moreover, to the best of our knowledge, we are the first to report a KIT mutation in exon 13 in dysgerminoma (Hersmus et al., 2012). Previous studies demonstrated that imatinib, a KIT gene inhibitor, led to a complete response of stage-IV chemoresistant seminoma (Pectasides et al., 2008), and PD-1 blockers significantly augmented the antitumor efficacy of imatinib (Seifert et al., 2017). Additionally, KIT mutations in exon 13 amplified the sensitivity of tumor cells to imatinib, sunitinib, and regorafenib, leading to prolonged survival in patients (McAuliffe et al., 2008; Napolitano and Vincenzi, 2019). Consequently, after primary radical surgery, combining tyrosine kinase inhibitors with ICIs or dysgerminoma-sensitized adoptive cellular therapy might provide more effective treatments to manage KIT mutation-associated dysgerminoma. Given the significant role of KIT mutations in cancer formation and development and their prevalence in dysgerminoma (Hersmus et al., 2012), it is critical to exclude KIT mutations in Turner syndrome patients, particularly those with Y mosaicism, to reduce the risk of dysgerminoma induced by hormone therapy.

Lastly, dysfunction in blood dynamics and abnormality of intracranial arteries extensively exist in Turner syndrome patients (Silberbach et al., 2018; Kruszka et al., 2019). Our patient also showed malformation of the circle of Willis (Figure 4). Considering that cisplatin-based chemotherapy is the standard treatment for dysgerminoma and is prone to induce hypercoagulability (De Giorgi et al., 2019), intracranial MRA becomes a crucial procedure in the evaluation of prognosis due to the high risk of thromboembolism. In this case, our patient exhibited normal coagulative parameters before chemotherapy, despite experiencing spontaneous intracranial embolism once. Following prophylactic anticoagulation with enoxaparin at a dose of 4,000 IU (40 mg) daily, multiple cerebral infarctions after chemotherapy were larger and more widespread than before (Figure 4). This indicates that selecting a moderate and sustained anticoagulation strategy, such as combining enoxaparin with *Ginkgo* leaf extract and dipyridamole (Liu et al., 2019), may provide more advantages to Turner syndrome patients undergoing cisplatin-based chemotherapy for dysgerminoma.

## Conclusion

In summary, Mullerian anomalies are not incompatible with Turner syndrome. Assessing KIT mutations is critical to Turner syndrome patients prior to considering further treatments. Comprehensive anticoagulation therapy is a pivotal procedure in the management of Turner syndrome patients undergoing cisplatin-based chemotherapy.

## Data Availability

The datasets presented in this article are not readily available because of ethical/privacy restrictions. Requests to access the datasets should be directed to the corresponding authors.

## Abbreviations

AFP, alpha fetal protein; CA-125, carbohydrate antigen 125; CA-19-9, carbohydrate antigen 19-9; CEA, carcinoembryonic antigen; CTL, cytotoxic T lymphocyte; ICI, immune checkpoint inhibitor; LDH, lactic dehydrogenase; LMWH, low-molecular-weight heparin; MRA, magnetic resonance angiography; MRI, magnetic resonance imaging; PD-1, programmed cell death protein 1; PDL-1, programmed death-ligand 1; TME, tumor microenvironment; β-hCG, beta human choriogonadotropin.
